# A Liver Transplant for Local Control in a Pediatric Patient with Metastatic TFE3-Associated Perivascular Epithelioid Cell Tumor (PEComa) to the Liver

**DOI:** 10.1155/2021/3924565

**Published:** 2021-10-05

**Authors:** Whayoung Lee, Josephine HaDuong, Aaron Sassoon, Tuan Dao, Ali Nael

**Affiliations:** ^1^Department of Pathology, University of California Irvine Medical Center, 101 The City Drive South, Bldg. 1, Room 3003 Orange, CA 92868, USA; ^2^Pediatric Oncology Department, Children's Hospital of Orange County (CHOC), 1201 West La Veta Ave. Orange, CA 92868, USA; ^3^Pathology Department, Children's Hospital of Orange County (CHOC), 1201 West La Veta Ave. Orange, CA 92868, USA; ^4^Radiology Department, Children's Hospital of Orange County (CHOC), 1201 West La Veta Ave. Orange, CA 92868, USA; ^5^Pathology Department, Children's Hospital of Orange County (CHOC) and University of California Irvine Medical Center, 1201 West La Veta Ave. Orange, CA 92868, USA

## Abstract

Perivascular epithelioid cell tumors (PEComas) are rare mesenchymal tumors with widespread distribution throughout the body and unpredictable clinical behavior. Recently, a subset of these tumors has been reported to harbor Transcription Factor E3 (TFE3) gene rearrangement with distinct morphologic and immunophenotypic features. Although limited, these tumors may represent a separate entity from the conventional PEComas and may require different treatment approaches. Surgery is the main treatment option with no clear consensus on systemic therapy. Here, we present the first case of a malignant pediatric colonic TFE3-associated PEComa with isolated liver metastasis leading to liver transplantation for the local control.

## 1. Introduction

Perivascular epithelioid cell tumors (PEComas) are rare mesenchymal tumors composed of perivascular epithelioid cells exhibiting melanocytic and muscular differentiation. The distribution is ubiquitous throughout the body, including skin, soft tissue, bone, and visceral organs [1]. Clinical behavior ranges from benign to malignant, without a reliable predictor. Surgery remains the mainstay of treatment with no clear consensus on systemic therapy for malignant or metastatic tumors. While mostly arising sporadically, a strong association with tuberous sclerosis complex (TSC) and a high frequency of TSC1 or TSC2 inactivation mutation has been reported [2, 3]. Recently, a subset of PEComas has been found to harbor Transcription Factor E3 (TFE3) gene rearrangement, which seems to be mutually exclusive to the TSC mutation [4–7]. Although cases are limited, distinct histologic and immunophenotypic features of TFE3-associated PEComas such as alveolar architecture composed of epithelioid cells with a clear and eosinophilic cytoplasm, no significant pleomorphism, strong TFE3 immunoreactivity, and absence of smooth muscle differentiation by immunohistochemical staining suggest that these may represent a separate entity [5, 7]. Here, we report the first case of a pediatric colonic TFE3-associated PEComa with isolated distant metastasis to the liver requiring a liver transplant to control the disease.

## 2. Case Presentation

**Figure 1:**
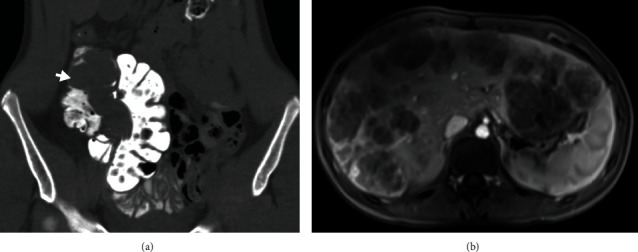
(a) Coronal CT image of the abdomen and pelvis with oral contrast demonstrates an intraluminal mass in the terminal ileum and ascending colon (arrow) serving as a lead point for ileocolic intussusception. (b) Axial T1 fat-saturated postcontrast MRI of the abdomen demonstrates multiple peripherally enhancing lesions throughout the liver consistent with liver metastasis.

**Figure 2:**
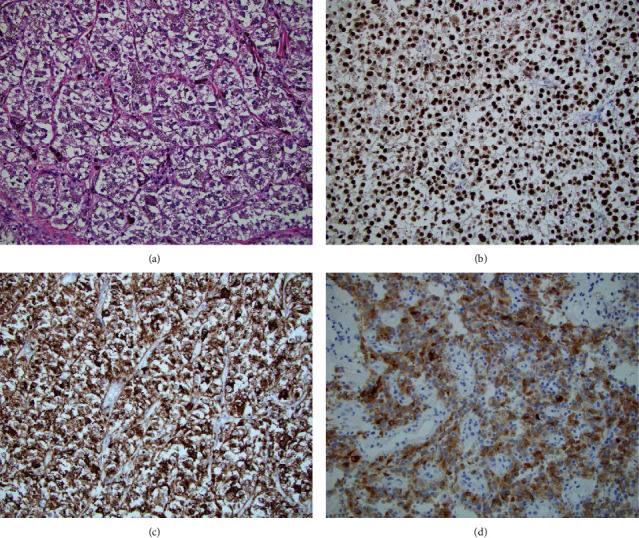
(a) The histologic features of the tumor which were composed of nests and sheets of epithelioid cells with frequent melanin pigmentation separated by thin-walled capillary vessels (predominant alveolar architecture) (hematoxylin and eosin (H&E) staining, magnification ×200). Tumor cells demonstrate immunohistochemical reactivity for (b) TFE3, (c) HMB45, and (d) Melan A (b–d, magnification ×200).

A sixteen-year old, otherwise, healthy female presented with 3 days of worsening abdominal pain. Abdomen/pelvic computed tomography (CT) demonstrated ileocecal intussusception, with a 5 cm mass as the lead point (Figure 1(a)). A diagnostic laparoscopy and subsequent right hemicolectomy were performed. The polypoid tumor was confined to the cecum with negative resection margins. Microscopically, the tumor was composed of nests and sheets of uniform epithelioid cells with a pale eosinophilic cytoplasm separated by thin-walled capillary vessels (Figure 2(a)). Tumor cells with melanin pigmentation and foci of tumor necrosis were seen. Mitotic figures were up to 9 per 10 high power fields (HPF), and lymphovascular invasion was identified. Thirty-nine lymph nodes were found without metastatic disease. By immunohistochemistry, the tumor cells were positive for HMB45, Melan A, TFE3 (Figures 2(b)–2(d)), and inhibin and negative for cytokeratin, PAX8, CD117, DOG1, smooth muscle actin, desmin, S100, and neuroendocrine markers. A fluorescence in situ hybridization (FISH) study confirmed TFE3 (Xp11.23) gene rearrangement with no evidence of ASPSCR1 gene fusion. The diagnosis of localized PEComa with a high-risk malignant potential was conferred. A follow-up image at 3 months postoperation detected metastatic recurrence diffusely involving but isolated to the liver (Figure 1(b)) that was confirmed by a core biopsy. She was started on sirolimus with no response and then transitioned to gemcitabine/docetaxel with no radiographic evidence of metastatic spread beyond the liver after 6 cycles. She received a donor liver 6 months after recurrence. The explant pathology showed the same tumor with negative resection margins, less than 50% necrosis, and eight hilar lymph nodes with tumor involvement. Although regional lymph node involvement was seen, posttransplant imaging has not detected recurrence and the patient remains disease free eight months posttransplant.

## 3. Discussion

As PEComas have been increasingly recognized in recent years, the biggest challenge is to predict the clinical behavior of the tumor. It ranges from benign to malignant, without an absolute correlation with the site of origin or definite histologic predictors. The World Health Organization (WHO) proposes the use of different histologic parameters (adopted from Folpe et al. [8]) for the identification of PEComas with a potentially aggressive clinical behavior, mainly applying to the PEComas in the gynecologic tract [6]. Those include size larger than 5 cm, infiltrative growth pattern, high nuclear grade and cellularity, mitoses greater than 1 per 50 HPF, necrosis, and vascular invasion [6]. A subsequent study by Bleeker et al. looking at 234 PEComas from different organs used the same WHO/Folpe parameters to divide these tumors into three categories: benign (<2 high-risk features and size < 5 cm), uncertain malignant potential (size ≥ 5 cm with no other high-risk features or nuclear pleomorphism alone), or malignant (2 or more high-risk features) [1]. Currently, the Folpe criteria are used for limited prediction of the tumor behavior, as only about half of the patients diagnosed as having “malignant” PEComa recur following resection [1, 8].

Recently, a subset of PEComas has been reported to harbor TFE3 gene rearrangement, a member of the MiT family of transcription factors. TFE3-associated PEComas have unique histologic and immunophenotypic features, including alveolar architecture composed of epithelioid cells with a clear and eosinophilic cytoplasm, no significant pleomorphism, strong TFE3 immunoreactivity, and absence of smooth muscle differentiation by immunohistochemical staining [5, 7, 9].

Approximately 50 cases of PEComas arising from the gastrointestinal (GI) tract have been reported in the literature. Like PEComas from other areas, GI PEComas exhibit a spectrum of biologic behavior from benign to malignant with no clear consensus on how to predict malignant behavior or optimal systemic therapy. Doyle et al. studied 35 GI PEComas and reported that approximately 30% of cases exhibited malignant behavior with local recurrence, distant metastases, or disease-related death [2]. Marked atypia, diffuse pleomorphism, and mitoses ≥ 2/10 HPF were significantly associated with the malignant behavior [2]. Unlike renal PEComas, GI PEComas are less likely associated with TSC mutations [4, 5].

Only 13 pediatric GI PEComas cases have been reported, ranging from 6 to 17 years of age with a female predilection, and the most common location is the distal colon (Table 1) [5, 10–21]. Interestingly, 2 cases occurred in patients with history of neuroblastoma, and no association with TSC mutations has been observed [12, 17]. TFE3 immunostaining was performed in four cases, and they were all positive. However, FISH confirmation was performed in two of them. Two cases presented with the regional lymph node involvement at the time of surgery (only one received chemotherapy). As opposed to GI PEComas in adults with 30% exhibiting malignant behavior, no disease-related death, distance metastasis, or local recurrence has been reported in the pediatric cases (except our case) with an average follow-up of 37 months, including the two cases with regional lymph node involvement at the time of diagnosis [5, 15–17].

While optimal treatment for PEComa has not been established, surgical resection with clear margin appears to be the mainstay of the therapy. Most patients who have progressive disease with distant metastases or lymph node involvement receive adjuvant treatment but the benefit has not yet been determined [1]. The fact that germline or sporadic TSC mutation results in subsequent activation of the mammalian target of rapamycin (mTOR) pathway supports the use of mTOR inhibitors (sirolimus) for targeted therapy in patients with locally aggressive or metastatic disease [2], which have been promising in some studies [1, 3, 6, 22]. As there is no TSC gene involvement in TFE3-associated PEComas, it is unclear if these patients would respond to mTOR inhibitors. However, targeting the MiT family of transcription factors where the TFE3 gene resides may aid the treatment of this distinct entity. Other agents exhibiting activity in PEComas include antiangiogenics and gemcitabine- or anthracycline-based chemotherapy regimens [22].

Given that PEComas cannot be successfully treated without surgery, our patient was considered for liver transplantation as definitive resection after demonstrating no extrahepatic disease for six months after recurrence. This represents the first case to our knowledge of a patient with a diagnosis of PEComa receiving a transplant, a resection option that may be considered in pediatric patients with the disease isolated to one organ.

Although it is a rare tumor, PEComa should be considered in the differential diagnosis of mesenchymal tumors, particularly of the GI tract, and TFE3 status should be evaluated in all cases, as TFE3-associated PEComas may represent as a distinct entity from conventional PEComas and may require different therapeutic decisions.

## Figures and Tables

**Table 1:** Graph demonstrating results.

Age (yr.)/sex	Location	Size (cm)	Margin status	Lymph node	Nuclear atypia	Mitosis/10 HPF	Prognosis^a^	TFE3 IHC^b^	TFE3 FISH^c^	Reference
6/F	Appendix	1.3	NA	NA	Yes	0	NA	NA	NA	[10]
9/F	Rectum	3	Neg	Neg	No	Rare	14 MO NED	NA	NA	[11]
12/M	Duodenum	3.5	Neg	Neg	No	Low	24 MO NED	NA	NA	[12]
16/F	Transverse colon	2	NA	Neg	NA	0	24 MO NED	NA	NA	[13]
11/M	Sigmoid colon	1.2	Neg	Pos	No	Occasional	5 MO NED	NA	NA	[14]
11/M	Descending/sigmoid colon	3.5	Neg	NA	NA	Infrequent	NA	Pos	NA	[15]
16/F	Transverse colon	1.8	NA	Neg	NA	NA	41 MO NED	Pos	NA	[16]
14/F	Sigmoid colon	6.4	Neg	NA	NA	Rare	NA	Pos	Pos	[17]
15/F	Rectum	3.7	Neg	Pos	No	2	9 MO NED	NA	NA	[18]
16/F	Colon	NA	NA	NA	NA	NA	NA	Pos	Pos	[5]
17/F	Sigmoid colon	6	Neg	NA	No	NA	180 MO NED	NA	NA	[19]
7/M	Ascending colon	4	Neg	Neg	No	Low	26 MO NED	NA	NA	[20]
17/M	Rectum	3	Neg	Neg	No	<1	10 MO NED	NA	NA	[21]
16/F	Ascending colon	5	Neg	Neg	No	Up to 9	Liver met. after 3 MO	Pos	Pos	Our case

Abbreviation: F: female; FISH: fluorescent in situ hybridization; HPF: high power fields; IHC: immunohistochemistry; M: male; Met: metastasis; MO: month; NA: no data available; Neg: negative; NED: no evidence of disease; Pos: positive; Ref.: reference; yr.: year(s). ^a^Follow-up since the first diagnosis. ^b^TFE3 expression by immunohistochemistry. ^c^Gene rearrangement involving TFE3 (Xp11.23) by FISH.
